# Catheter-Directed Therapies in Patients with Pulmonary Embolism: Predictive Factors of In-Hospital Mortality and Long-Term Follow-Up

**DOI:** 10.3390/jcm10204716

**Published:** 2021-10-14

**Authors:** Jesús Ribas, Joana Valcárcel, Esther Alba, Yolanda Ruíz, Daniel Cuartero, Adriana Iriarte, José María Mora-Luján, Marta Huguet, Pau Cerdà, Sergio Martínez-Yélamos, Xavier Corbella, Salud Santos, Antoni Riera-Mestre

**Affiliations:** 1 Pneumology Department, Hospital Universitari de Bellvitge, 08907 Barcelona, Spain; yolanda.ruiz@bellvitgehospital.cat (Y.R.); ssantosp@bellvitgehospital.cat (S.S.); 2 Bellvitge Biomedical Research Institute (IDIBELL), 08907 Barcelona, Spain; jvalcarcel@bellvitgehospital.cat (J.V.); estheralba@bellvitgehospital.cat (E.A.); dcuartero@bellvitgehospital.cat (D.C.); adriana.iriarte@bellvitgehospital.cat (A.I.); jmora@bellvitgehospital.cat (J.M.M.-L.); mhuguet@bellvitgehospital.cat (M.H.); pcerda@idibell.cat (P.C.); smartinezy@bellvitgehospital.cat (S.M.-Y.); xcorbella@bellvitgehospital.cat (X.C.); ariera@bellvitgehospital.cat (A.R.-M.); 3 Biomedical Research Networking Center on Respiratory Diseases (CIBERES), 28029 Madrid, Spain; 4 Radiology Department, Hospital Universitari de Bellvitge, 08907 Barcelona, Spain; 5 Internal Medicine Department, Hospital Universitari de Bellvitge, 08907 Barcelona, Spain; 6 Critical Care Medicine Department, Hospital Universitari de Bellvitge, 08907 Barcelona, Spain; 7 Neurology Department, Hospital Universitari de Bellvitge, 08907 Barcelona, Spain; 8 Faculty of Medicine and Health Sciences, Universitat de Barcelona, L’Hospitalet de Llobregat, 08907 Barcelona, Spain; 9 Faculty of Medicine and Health Sciences, Universitat Internacional de Catalunya, 08017 Barcelona, Spain

**Keywords:** endovascular procedures, mortality, pulmonary embolism, thrombectomy, thrombolytic therapy, vena cava filters

## Abstract

(1) Background: Catheter-directed therapies (CDT) may be considered for selected patients with pulmonary embolism (PE); (2) Methods: Retrospective observational study including all consecutive patients with acute PE undergoing CDT (mechanical or pharmacomechanical) from January 2010 through December 2020. The aim was to evaluate in-hospital and long-term mortality and its predictive factors; (3) Results: We included 63 patients, 43 (68.3%) with high-risk PE. All patients underwent mechanical CDT and, additionally, 27 (43%) underwent catheter-directed thrombolysis. Twelve (19%) patients received failed systemic thrombolysis (ST) prior to CDT, and an inferior vena cava (IVC) filter was inserted in 28 (44.5%) patients. In-hospital PE-related and all-cause mortality rates were 31.7%; 95% CI 20.6–44.7% and 42.9%; 95% CI 30.5–56%, respectively. In multivariate analysis, age > 70 years and previous ST were strongly associated with PE-related and all-cause mortality, while IVC filter insertion during the CDT was associated with lower mortality rates. After a median follow-up of 40 (12–60) months, 11 more patients died (mortality rate of 60.3%; 95% CI 47.2–72.4%). Long-term survival was significantly higher in patients who received an IVC filter; (4) Conclusions: Age > 70 years and failure of previous ST were associated with mortality in acute PE patients treated with CDT. In-hospital and long-term mortality were lower in patients who received IVC filter insertion.

## 1. Introduction

Pulmonary embolism (PE) is among the most common causes of vascular mortality after myocardial infarction and stroke, being the leading preventable cause of death in hospitalized patients [1]. Shock and systemic hypotension identify those PE patients at high risk of mortality [2]. Systemic thrombolysis (ST) is recommended for high-risk PE patients, aiming to rapidly reverse hemodynamic compromise by relieving right ventricle (RV) pressure overload, thus reducing the probability of death due to RV failure [3,4,5,6]. Among patients with intermediate-risk PE, up to 5% may experience hemodynamic decompensation on anticoagulation, and rescue ST is also recommended for these patients [3,4,5,7].

Due to increased risk for major bleeding (MB) with no evidence of reduction in mortality, ST is not recommended for most normotensive PE patients [8]. In fact, guidelines recommended the use of ST for (1) patients with acute symptomatic PE and hemodynamic instability who do not have major contraindications owing to bleeding risk, and (2) patients without hypotension who experience hemodynamic deterioration while receiving anticoagulant therapy [4]. Despite these recommendations, ST is an underused treatment, with some studies indicating that only 30% of high-risk PE patients receive it [9,10]. Concern about MB and intracranial hemorrhage could be among the main factors responsible for this underuse [11].

Catheter-directed therapies (CDT) are currently considered a treatment option for selected patients with PE [12]. CDT have acquired relevance because of the limitations of both anticoagulation and ST in patients with a high bleeding risk and the complexity and risks of surgical embolectomy [13]. Overall, CDT attempt to rapidly decrease thrombus burden and restore hemodynamic normality through mechanical (fragmentation and aspiration), pharmacological (local thrombolysis) or the combination of both procedures [13]. CDT have the potential to offer a clinical benefit similar to ST with a lower expected risk of MB [14]. According to guidelines, CDT should be considered for patients with high-risk PE in whom ST is contraindicated or has failed [3,4,5]. Most knowledge about CDT is derived from studies mostly including intermediate-risk PE patients with low mortality rates, and predictors of in-hospital mortality are rarely reported [15,16,17,18,19,20,21,22]. Moreover, little is known on the long-term survival of PE patients undergoing CDT [23,24]. We aimed to assess in-hospital and long-term mortality in a series of patients with acute PE undergoing CDT.

## 2. Materials and Methods

### 2.1. Study Design and Patient Selection

Retrospective observational study carried out in a university hospital during the period January, 2010, to December, 2020. Our hospital is a referral center for highly technical processes for more than 2 million inhabitants in the southern part of Catalonia (Spain). All consecutive patients with acute objectively confirmed PE who underwent CDT (mechanical or pharmacomechanical) during the study period were included. Patients were identified from the database of the Interventional Vascular Radiology Unit, which performs all CDT procedures in our hospital. Therefore, the study includes all PE patients treated with CDT during the study period.

The primary objective was to assess in-hospital PE-related mortality and its predictive factors in a series of patients with acute PE undergoing CDT. Secondary objectives were to assess all-cause in-hospital mortality and long-term outcomes.

Data were obtained from routine daily practice and anonymized to a secure database. Personal and clinical collected data were in line with the Spanish Data Protection Act (Ley Orgánica 3/2018 de 5 de diciembre de Protección de Datos PersonalesWe followed the Strengthening the Reporting of Observational Studies in Epidemiology (STROBE) statement guidelines for observational cohort studies [25].

### 2.2. Variables and Outcome Measures

Extracted data included patient demographics, history of venous thromboembolism (VTE), active cancer (defined as newly diagnosed cancer or cancer undergoing treatment), cardiovascular risk factors, chronic heart, renal or lung disease, recent bleeding (<30 days before PE), surgery in the previous 2 months, residual deep venous thrombosis (DVT), reason for hospital admission and need for intensive care unit (ICU) admission. D-dimer and troponin blood tests were collected. Bedside transthoracic echocardiography to assess RV dilation, computed tomography (CT) features of PE (bilaterality, most proximal vessel involved, RV dilation according to RV/left ventricle ratio >1 and the CT-derived Qanadli index) and stratification using the simplified Pulmonary Embolism Severity Index (sPESI) were collected [26,27].

The hemodynamic status was evaluated by systolic blood pressure (BP) and by the shock index (heart rate/systolic BP). As a measure of gas-exchange status we calculated the ratio of oxygen saturation by pulse oximetry/fraction of inspired oxygen (SpO_2_/FiO_2_). High-risk PE was defined as acute PE with systolic BP < 90 mmHg, requiring inotropic support or presenting as cardiac arrest. Patients who did not meet the high-risk criteria were classified as intermediate-risk PE according to the ESC Guidelines [5].

Mortality was assessed at two time points: in-hospital and during follow-up. Deaths following a clinically severe PE, in the absence of any alternative diagnosis, were classified as fatal PE. Bleeding risk was assessed retrospectively by means of the BACS score and patients were classified as high, intermediate or low-risk categories [28]. MB and clinically relevant non-MB were defined according to the ISTH criteria [29,30].

### 2.3. Catheter-Directed Therapy

Patients were managed according to the clinical practice in our hospital as decided by the attending physicians. Briefly, CDT was indicated for patients in whom ST was contraindicated or had failed and for patients with features suggesting an adverse clinical outcome (i.e., new hemodynamic instability or worsening respiratory failure).

Vascular access was obtained by placing two 8-F (ipsilateral) or a single 10-F vascular sheath in the common femoral vein. The main pulmonary artery was catheterized by using a 5-F angled pigtail diagnostic catheter, and an initial pulmonary angiogram was obtained. Mechanical CDT (fragmentation and/or aspiration) was performed in all patients and catheter-directed thrombolysis (CDL) was administered at the discretion of the interventional radiologist. In some patients, hemodynamic measurements were obtained pre and post-procedure. The procedure was terminated when the interventional radiologist considered that the thrombus burden had been reduced as much as possible or patient’s hemodynamic stability was achieved. An inferior vena cava (IVC) filter was placed in selected cases during the CDT at the discretion of the treating medical team. After completion of CDT, all patients without contraindications resumed anticoagulation.

### 2.4. Patients’ Follow-Up

After hospital discharge, VTE recurrence, residual pulmonary obstruction (RPO), development of chronic thromboembolic pulmonary hypertension (CTEPH) and mortality were collected during follow-up. Vital status at 28 February 2021 was recorded. The presence of RPO was assessed by means of pulmonary CT angiography or perfusion lung scan. CTEPH was diagnosed according to guidelines [31].

### 2.5. Statistical Analysis

Categorical variables are expressed as frequencies and proportions. Continuous variables are expressed as means with standard deviations (SD) or medians with interquartile range (IQR). For quantitative variables, comparisons between groups of patients were performed using the unpaired Student *t*-test. Categorical variables were compared with the Chi-square test (or with Fisher’s exact test when appropriate). The Kaplan–Meier method was used to estimate overall survival in the whole group of patients, with censoring at the date of last follow-up. Comparison between groups was assessed with the Log-rank test.

Univariate analyses were performed to select predictive variables of mortality for the multivariate model. Selection of variables included in the multivariate model was based on clinical (known risk factors for in-hospital mortality) and statistical significance (variables that were associated with in-hospital mortality on univariate analysis with a *p* value < 0.20). Missing data were not imputed for the multivariate analyses. We used a stepwise logistic regression model with forward selection to determine the contribution of all candidate covariates, with a threshold for candidate inclusion of ≤0.05 and for elimination of ≥0.10. Odds ratios and 95% confidence intervals (CIs) were used to quantify the association. A *p*-value of <0.05 was considered statistically significant. Analyses were performed using IBM SPSS Statistics (version 19.0, Armonk, NY, USA) for the PC.

## 3. Results

### 3.1. Clinical Baseline Characteristics at PE Presentation

During the study period, 63 patients with acute PE underwent CDT, 43 (68.3%) with high-risk PE. Mean age was 60.2 ± 13.9 years and 32 (50.8%) were men. Fourteen (22.2%) patients were >70 years old. Twelve (19%) patients had history of VTE and 16 (25.4%) had active cancer. Residual DVT was observed in 72.3% of patients. Overall, 22 (34.9%) patients had recent bleeding and/or surgery. The most frequent reason for hospital admission was acute PE (71.4%). Fifty-four (85.7%) patients were admitted to the ICU. Clinical presentation of PE was different in high-risk PE patients compared to intermediate-risk patients. The rest of clinical baseline characteristics were similar between these two groups (Table 1).

### 3.2. Characteristics of Acute PE and Treatment

Overall, our study population had an unfavorable hemodynamic profile with a median systolic BP of 90 (80–120) mmHg and a median shock index of 1.15 (0.93–1.41), more severe in high-risk patients. Regarding gas-exchange, the SpO_2_/FiO_2_ ratio showed no significant differences between high and intermediate-risk patients. Plasma troponin levels were positive in 77% of patients in which it was assessed, more frequently in high-risk patients. On chest CT scan, most patients had bilateral PE with thrombi at the main pulmonary artery level, with RV dilation in 68% of patients. The degree of pulmonary vascular obstruction was not different between both groups. Bedside echocardiography was performed at PE diagnosis in 48 (76.2%) patients, showing RV dilation in 98% of them, with a mean systolic pulmonary artery pressure (PAP) of 60 ± 12 mmHg and a mean tricuspid annular plane systolic excursion of 14.3 ± 5.2 mm. Hemodynamic measurements were obtained pre-procedure in 44 (70%) patients, showing moderate pulmonary hypertension (Table 2).

All patients underwent mechanical CDT and, additionally, 27 (43%) of them underwent CDL. Urokinase with a median dose of 490,000 (300,000–2,400,000) IU was administered in 14 (52%) patients and recombinant tissue plasminogen activator with a median dose of 70 (31–90) mg in the remaining 13 (48%) patients. The proportion of patients receiving CDL did not show differences between high and intermediate-risk patients. When comparing patients who underwent isolated mechanical CDT (*n* = 36) with patients who underwent CDL added to mechanical CDT (*n* = 27), the former had a lower frequency of arterial hypertension and a higher frequency of previous VTE (Supplementary Table S1).

Twelve (19%) patients were treated with ST before the CDT, of which six (9.5%) also received CDL. Thirty-two (50.8%) patients had both invasive mean PAP measures before and after the CDT, showing a significant reduction, from 37 ± 10 mmHg to 33 ± 9 mmHg (*p* = 0.001). An IVC filter was inserted in 28 (44.5%) patients during the CDT procedure. Indications for filter insertion were: contraindication to anticoagulation in 12 (42.9%) patients and high-risk PE in 16 (57.1%) patients (Table 2).

When comparing patients with (*n* = 28) and without (*n* = 35) filter insertion, the former had a lower frequency of chronic renal failure and a higher frequency of recent surgery. The most frequent clinical presentation of PE among patients with filter insertion was dyspnoea ± chest pain, while only 10.7% of patients in this group presented as cardiac arrest (Supplementary Table S2). 

### 3.3. In-Hospital Outcomes

A MB episode occurred in eight (12.7%; 95% CI 5.6–23.5%) patients and a clinically relevant non-MB episode occurred in three (4.8%; 95% CI 1.0–13.3%) patients. The sites of MB were hemoptysis in 3 (37.5%) patients, gastrointestinal in 2 (25%), retroperitoneal in two (25%) and soft tissue in one (12.5%) patient. Among patients with MB, five (62.5%) had undergone CDL and three (37.5%) had not (*p* = 0.272). The BACS score was different between both groups, with more patients in the high category among high-risk PE patients (Table 2). All 12 patients who received ST before CDT (including six receiving CDL) had a low or intermediate risk of bleeding, and none of them had MB. Although not reaching statistical significance, we observed a linear association between the stages of the BACS score and a growing frequency of MB (Table 3). No patient developed intracranial bleeding and there was no fatal bleeding during hospitalization.

**Figure 1:**
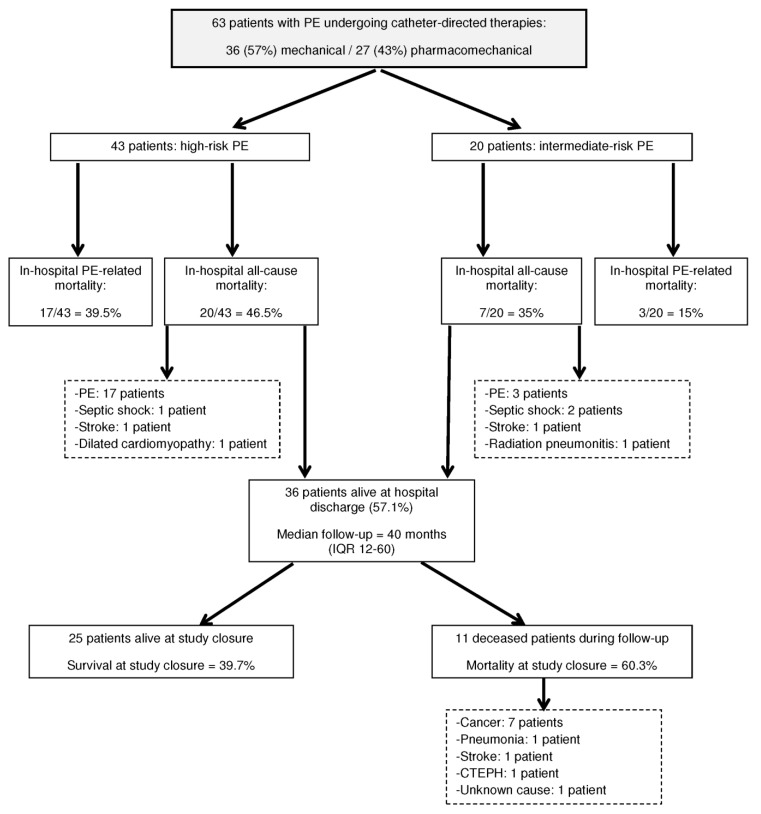
Diagram showing mortality distribution throughout the study. Abbreviations: PE, pulmonary embolism; IQR, interquartile range; CTEPH, chronic thromboembolic pulmonary hypertension.

The mortality distribution throughout the study is shown in Figure 1. In-hospital PE-related mortality rate was 31.7%; 95% CI 20.6–44.7%, being significantly higher among high-risk patients (*p* = 0.051) (Table 2). In univariate analysis, age > 70 years, diabetes, chronic renal failure, presentation of PE as cardiac arrest, and previous ST were associated with a greater mortality risk. IVC filter insertion showed a protective mortality effect. In multivariate analysis, age > 70 years and previous ST were strongly associated with PE-related mortality, while IVC filter insertion was associated with lower mortality rates (Table 4).

In-hospital all-cause mortality rate was 42.9%; 95% CI 30.5–56%, without significant differences between high and intermediate-risk patients (*p* = 0.390) (Table 2). The causes of death in the seven non-PE-deceased patients were: septic shock (3 patients), stroke (2 patients), cardiogenic shock (1 patient), and radiation pneumonitis (1 patient). In univariate analysis, age > 70 years, diabetes, chronic renal failure and previous ST were also associated with a greater mortality risk and IVC filter insertion showed also a protective effect. In multivariate analysis, we observed the same pattern of association of variables with mortality as in PE-related mortality (Table 5).

It is worth mentioning that CDL was not significantly associated with PE-related or all-cause mortality in univariate analysis. Consequently, this variable was not included in the multivariate models.

### 3.4. Long-Term Follow-Up

**Figure 2:**
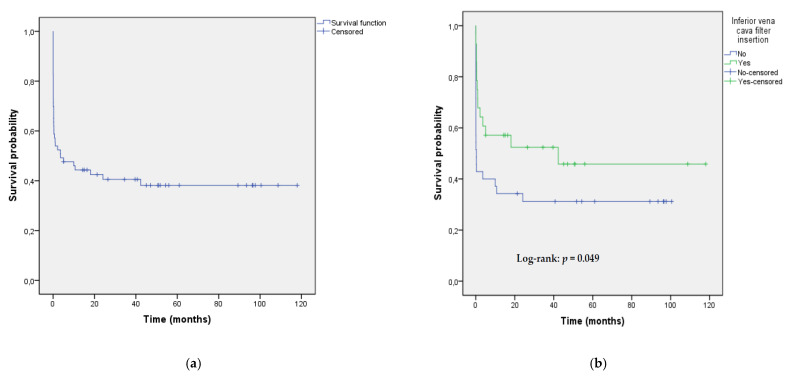
(**a**) Kaplan–Meier survival estimates from the time of endovascular reperfusion therapy for the whole group of 63 patients with pulmonary embolism (20-month, 40-month and 100-month survival estimates were 0.42, 0.40 and 0.38, respectively); (**b**) Kaplan–Meier survival estimates from the time of endovascular reperfusion therapy stratified by inferior vena cava filter insertion.

Thirty-six (57.14%) patients were discharged from hospital. After a median follow-up of 40 (12–60) months, 11 patients died, the most frequent cause of death was cancer (Figure 1). Accordingly, the mortality rate was 60.3% (95% CI 47.2–72.4%). Median survival time was 3.6 (95% CI, 0–14.3) months for the whole group of 63 patients, and Kaplan–Meier 20-month, 40-month and 100-month survival estimates were 0.42, 0.40 and 0.38, respectively (Figure 2a). There was a significant difference in survival between patients with IVC filter insertion versus patients without filter insertion (Figure 2b).

Among patients discharged from the hospital, four (11%) had VTE recurrence during follow-up (DVT in two patients and PE in two patients). After a median follow-up of 9.6 (6.2–17.7) months, 25 (69%) patients underwent imaging follow-up tests, showing RPO in nine (36%). One (2.78%; 95% CI 0.1–14.5%) patient developed CTEPH during follow-up.

## 4. Discussion

In this real-world study, we have reported the mortality and long-term outcomes in a series of mostly high-risk PE patients treated with CDT, providing new information on predictive factors of mortality. In these patients, age > 70 years and previous ST are risk factors for mortality, while IVC filter insertion during the CDT is associated with lower mortality rates. The in-hospital mortality rate in our study was high and mainly PE-related, a fact that could be justified by the severity of the clinical–radiological presentation. Our results are in line with previous studies, which have reported mortality rates in high-risk PE patients ranging between 22 and 58% [2,32].

Few studies have addressed late clinical outcomes after CDT. In a recent study that included 15 patients undergoing aspiration thrombectomy for high-risk PE, the 5-year survival rate was 75%, with cancer being the most common cause of death [23]. In another study including 48 patients with high-risk PE treated with rescue CDT following unsuccessful ST, after a mean follow-up of 2.8 ± 1.1 years, there was only one cancer-related death [24]. In our study, cancer was the most frequent cause of death during follow-up, adding valuable information on the long-term mortality of patients undergoing CDT. RPO rate and CTEPH incidence during follow-up in our study are in line with previously published data [5,33].

Only one risk score has been derived to predict major bleeding in PE patients treated with ST [28]. We retrospectively assessed bleeding risk by BACS score to test its potential usefulness in patients undergoing CDT. In our population of PE patients undergoing CDT (19% of them receiving ST prior to the CDT), we observed a linear association that approached statistical significance between BACS score’s categories and MB. Overall, ST was performed without MB events in patients classified as having low and intermediate risk of bleeding, and none of the six patients treated with both ST and CDL developed MB. These data suggest that the BACS score could also be useful for patients undergoing CDT, a hypothesis that should be validated in prospective studies.

Modern CDT are relatively safe and effective treatments for patients with acute high-risk PE, with pooled clinical success rates above 80% [34]. In our study, age > 70 years and treatment with ST before the CDT were strongly associated with mortality. Age is a known risk factor for mortality in patients with acute PE, being included in clinical scores for the assessment of PE severity [2,27,35]. Unsuccessful ST has been reported in up to 8% of patients with high-risk PE undergoing this therapy, leading to significant mortality [36]. According to our results, the combination of advanced age and ST failure could select a PE population with a particularly high mortality risk.

The most accepted indication for IVC filter insertion is the prevention of PE in patients with acute VTE and an absolute contraindication to anticoagulation [3,5,37]. There is uncertainty about the benefit of IVC filter insertion as an adjunct to ST in high-risk PE patients. However, IVC filter insertion has been associated with improved mortality outcomes in patients undergoing ST [9]. The rate of IVC filter insertion in patients undergoing CDT is variable and its influence on mortality has not been assessed [15,16,18,21,24,38]. Interestingly, we have identified that patients undergoing CDT in whom an IVC filter was inserted during the same procedure, showed a significant decrease in in-hospital mortality compared to patients without filter insertion. The prevention of PE recurrence in patients with severe acute PE undergoing CDT with poor cardiopulmonary reserve could account for this favorable effect on mortality. In fact, among patients with hemodynamic compromise in the International Cooperative Pulmonary Embolism Registry, insertion of an IVC filter was associated with a reduction of early recurrent PE and death [39].

Our study has some limitations and strengths that should be mentioned. The retrospective nature of the study and the lack of a control group are the main limitations. We addressed these limitations at least partially by performing multivariate analysis. However, with residual confounding being likely, our results are at best hypothesis generating. Moreover, due to the severity of clinical presentation, our study has some missing data on variables of recognized importance in PE patients (i.e., residual DVT). Regarding the apparent protective effect of IVC insertion, we cannot completely rule out the existence of a selection bias due to the fact that IVC filters were inserted preferentially in patients with a more favorable prognosis. However, the relatively high number of consecutive real-world patients included (most of them with high-risk PE) enhances the ability of our study to reflect the wide spectrum of patients who require CDT in daily clinical practice. Moreover, the existence of follow-up in all survivors provides knowledge about the clinical impact of CDT on long-term survival and the development of CTEPH.

## 5. Conclusions

In severe acute PE patients undergoing CDT, age > 70 years and failure of previous ST are associated with higher in-hospital mortality. In-hospital and long-term mortality were significantly lower in patients who received IVC filter insertion. The benefit of IVC filter insertion in patients undergoing CDT should be addressed in prospective randomized studies.

## Figures and Tables

**Table 1:** Clinical baseline characteristics at pulmonary embolism presentation.

Characteristics	Overall (*n* = 63)	High-Risk PE (*n* = 43)	Intermediate-Risk PE (*n* = 20)
Demographics			
Age, mean (SD)	60.2 (13.9)	61.6 (14.5)	57.3 (12.4)
Age > 70 years, *n* (%)	14 (22.2%)	12 (27.9%)	2 (10%)
Male gender, *n* (%)	32 (50.8%)	22 (51.2%)	10 (50%)
Underlying conditions			
Arterial hypertension, *n* (%)	22 (34.9%)	15 (34.9%)	7 (35%)
Diabetes, *n* (%)	7 (11.1%)	6 (14%)	1 (5%)
Chronic heart disease, *n* (%)	3 (4.8%)	2 (4.7%)	1 (5%)
Chronic lung disease, *n* (%)	11 (17.5%)	7 (16.3%)	4 (20%)
Chronic renal failure, *n* (%)	9 (14.3%)	7 (16.3%)	2 (10%)
Recent bleeding (<30 days), *n* (%)	8 (12.7%)	7 (16.3%)	1 (5%)
Risk factors for VTE			
Previous VTE, *n* (%)	12 (19%)	6 (14%)	6 (30%)
Active cancer, *n* (%)	16 (25.4%)	10 (23.3%)	6 (30%)
Recent surgery (<2 months), *n* (%)	17 (27%)	9 (20.9%)	8 (40%)
Reasons for hospital admission, *n* (%)			
PE	45 (71.4%)	31 (72%)	14 (70%)
Stroke	3 (4.8)	3 (7%)	0 (0%)
Medical conditions other than VTE	6 (9.5%)	3 (7%)	3 (15%)
Surgical procedures	6 (9.5%)	3 (7%)	3 (15%)
Polytrauma	3 (4.8%)	3 (7%)	0 (0%)
Length of hospital stay (days), Median (IQR)	13 (3–22)	12 (1–21)	13 (8–22)
Clinical presentation of PE, *n* (%)			
Dyspnea ± chest pain	30 (47.6%)	20 (46.5%)	10 (50%)
Syncope	19 (30.2%)	9 (20.9%)	10 (50%)
Cardiac arrest	14 (22.2%)	14 (32.6%)	0 (0%)

Abbreviations: PE, pulmonary embolism; VTE, venous thromboembolism; IQR, interquartile range.

**Table 2:** Pulmonary embolism characteristics, treatment and in-hospital mortality.

Characteristics	Overall (*n* = 63)	High-Risk PE (*n* = 43)	Intermediate-Risk PE (*n* = 20)
Simplified PESI Index ≥ 1, *n* (%)	61 (96.8%)	43 (100%)	18 (90%)
Hemodynamic status			
Systolic BP (mmHg), median (IQR)	90 (80–120)	82 (77–90)	120 (108–125)
Heart rate (beats/min), median (IQR)	114 (100–120)	117 (97–126)	112 (100–120)
Shock Index ^†^, median (IQR)	1.15 (0.93–1.41)	1.33 (1.07–1.5)	0.94 (0.82–1.1)
SpO_2_/FiO_2_ ratio, median (IQR)	371 (231–429)	371 (214–429)	367 (253–438)
Residual DVT ^§^, *n* (%)	34 (72.3%)	19 (63.3%)	15 (88.2%)
D-dimer µg/L, median (IQR)	5962 (3314–17,563)	7106 (3259–38,563)	5583 (3646–6160)
Positive troponin levels ^‡^, *n* (%)	40 (76.9%)	33 (91.7%)	7 (43.8%)
CT features			
Bilateral PE, *n* (%)	61 (96.8%)	42 (97.7%)	19 (95%)
PE at main PA level, *n* (%)	56 (88.9%)	39 (95.1%)	17 (85%)
RV/LV ratio on CT > 1, *n* (%)	43 (68.3%)	32 (82.1%)	11 (55%)
Qanadli Index (%), mean (SD)	37.7 (±14)	38 (±13)	37 (±16.9)
Echocardiographic features ^#^			
RV dilation, *n* (%)	48 (98%)	34 (100%)	14 (93.3%)
TAPSE (mm), mean (SD)	14.3 (5.2)	14.6 (5.1)	13.9 (5.6)
Systolic PAP (mmHg), mean (SD)	60 (12)	59 (12)	60 (13)
Bleeding risk (BACS score), *n* (%)			
Low	25 (39.7%)	20 (46.5%)	5 (25%)
Intermediate	30 (47.6%)	16 (37.2%)	14 (70%)
High	8 (12.7%)	7 (16.3%)	1 (5%)
Previous systemic thrombolysis, *n* (%)	12 (19%)	11 (25.6%)	1 (5%)
Mean PAP (mmHg) ^¶^ before CDT, mean (SD)	36 (±10)	36 (±10)	36 (±11)
CDL, *n* (%)	27 (42.9%)	18 (41.9%)	9 (45%)
IVC filter insertion, *n* (%)	28 (44.5%)	16 (37.2%)	12 (60%)
Major bleeding, *n* (%)	8 (12.7%)	6 (14%)	2 (10%)
In-hospital PE-related mortality, *n* (%)	20 (31.7%)	17 (39.5%)	3 (15%)
In-hospital all-cause mortality, *n* (%)	27 (42.9%)	20 (46.5%)	7 (35%)

^†^ Heart rate/systolic blood pressure; ^§^ Residual DVT was studied in 47 patients; ^‡^ Troponin was assessed in 52 patients; ^#^ Bedside echocardiography was performed in 48 patients; ^¶^ Mean PAP was measured in 44 patients. Abbreviations: PE, pulmonary embolism; BP, blood pressure; IQR, interquartile range; SpO2/FiO2, oxygen saturation by pulse oximetry/fraction of inspired oxygen; DVT, deep venous thrombosis; CT, computed tomography; PA, pulmonary artery; RV, right ventricle; LV, left ventricle; TAPSE, tricuspid annular plane systolic excursion; PAP, pulmonary artery pressure; CDL, catheter-directed thrombolysis; IVC, inferior vena cava.

**Table 3:** Major bleeding rates according to BACS score risk stratification.

Bleeding Risk, *n* (%)	Major Bleeding		
	Yes	No	
Low	1 (4%)	24 (96%)	*p*-value = 0.077 *
Intermediate	5 (16.7%)	25 (83.3%)	
High	2 (25%)	6 (75%)	

* Mantel-Haenszel linear-by-linear association.

**Table 4:** Factors associated with in-hospital PE-related mortality.

Risk Factor	Univariate Analysis		Multivariate Analysis	
	OR (95% CI)	*p*-Value	OR (95% CI)	*p*-Value
Age > 70 years	6.22 (1.72–22.43)	0.005	8.93 (1.58–50.34)	0.013
Sex (male)	1.73 (0.59–5.06)	0.321		
Active cancer	0.41 (0.10–1.63)	0.205		
Diabetes	6.83 (1.20–39.06)	0.031		
Chronic renal failure	5.71 (1.26–25.96)	0.024		
Clinical presentation of PE *		0.06		
Dyspnea ± chest pain				
Syncope	1.85 (0.49–6.89)	0.362		
Cardiac arrest	5.33 (1.33–21.33)	0.018		
High-risk PE	3.71 (0.94–14.60)	0.061		
Simplified PESI Index, per point	1.59 (0.95–2.65)	0.076		
Previous systemic thrombolysis	6.50 (1.66–25.41)	0.007	13.12 (1.84–93.75)	0.010
IVC filter insertion	0.073 (0.015–0.354)	0.001	0.06 (0.01–0.37)	0.002
CDL	0.619 (0.207-1.855)	0.392		

* Coded with respect to “Dyspnea ± chest pain” (reference category). Abbreviations: PE, pulmonary embolism; IVC, inferior vena cava; CDL, catheter-directed thrombolysis.

**Table 5:** Factors associated with in-hospital all-cause mortality.

Risk Factor	Univariate Analysis		Multivariate Analysis	
	OR (95% CI)	*p*-Value	OR (95% CI)	*p*-Value
Age > 70 years	4.71 (1.28–17.27)	0.020	4.83 (1.15–20.26)	0.031
Sex (male)	1.40 (0.51–3.81)	0.513		
Active cancer	0.52 (0.16–1.72)	0.282		
Diabetes	10.0 (1.13–88.91)	0.039		
Chronic renal failure	5.95 (1.13–31.47)	0.036		
High-risk PE	1.62 (0.54–4.84)	0.392		
Simplified PESI Index, per point	1.13 (0.72–1.79)	0.594		
Previous systemic thrombolysis	5.50 (1.32–22.92)	0.019	6.37 (1.31–30.82)	0.021
IVC filter insertion	0.250 (0.084–0.741)	0.012	0.28 (0.08–0.91)	0.034
CDL	0.500 (0.178-1.405)	0.188		

Abbreviations: PE, pulmonary embolism; IVC, inferior vena cava; CDL, catheter-directed thrombolysis.

## Supplementary Materials

The following are available online at https://www.mdpi.com/article/10.3390/jcm10204716/s1, Table S1: Comparison of clinical baseline characteristics at PE presentation between patients undergoing and not undergoing CDL in addition to mechanical CDT; Table S2: Comparison of clinical baseline characteristics at PE presentation between patients with and without inferior vena cava filter insertion.
