# Supplementary material for: Catheter-Directed Therapies in Patients with Pulmonary Embolism: Predictive Factors of In-Hospital Mortality and Long-Term Follow-Up
Source: J Clin Med. 2021 Oct 14;10(20):4716. doi: 10.3390/jcm10204716 (PMC8537142; doi:10.3390/jcm10204716)
Supplement: Supplementary file 1 [file jcm-10-04716-s001.zip › jcm-1390983-supplementary.pdf]

Table S1. Comparison of clinical baseline characteristics at PE presentation between patients undergoing and not undergoing CDL in addition to mechanical CDT

**Characteristics**	**CDL (*n* = 27)**	**No CDL (*n* = 36)**	***p*-Value**
Demographics			
Age, mean (SD)	59.5 (13.3)	60.8 (14.5)	0.731
Age > 70 years, *n* (%)	5 (18.5%)	9 (25%)	0.540
Male gender, *n* (%)	13 (48.1%)	19 (52.8%)	0.716
Underlying conditions			
Arterial hypertension, *n* (%)	14 (51.9%)	8 (22.2%)	0.015
Diabetes, *n* (%)	4 (14.8%)	3 (8.3%)	0.449
Chronic heart disease, *n* (%)	3 (11.1%)	0 (0%)	0.074
Chronic lung disease, *n* (%)	5 (18.5%)	6 (16.7%)	1
Chronic renal failure, *n* (%)	4 (14.8%)	5 (13.9%)	1
Recent bleeding (<30 days), *n* (%)	2 (7.4%)	6 (16.7%)	0.448
Risk factors for VTE			
Previous VTE, *n* (%)	2 (7.4%)	10 (27.8%)	0.042
Active cancer, *n* (%)	6 (22.2%)	10 (27.8%)	0.616
Recent surgery (<2 months), *n* (%)	5 (18.5%)	12 (33.3%)	0.190
High-risk PE, *n* (%)	18 (66.7%)	25 (69.4%)	0.815
Clinical presentation of PE, *n* (%)			0.996
Dyspnoea ± chest pain	13 (48.1%)	17 (47.2%)	
Syncope	8 (29.6%)	11 (30.6%)	
Cardiac arrest	6 (22.2%)	8 (22.2%)	

Abbreviations: PE, pulmonary embolism; CDL, catheter-directed thrombolysis; CDT, catheter-directed therapy; VTE, venous thromboembolism.

Table S2. Comparison of clinical baseline characteristics at PE presentation between patients with and without inferior vena cava filter insertion

Characteristics	Filter Insertion (*n* = 28)	No Filter Insertion (*n* = 35)	*p*-Value
Demographics			
Age, mean (SD)	58.5 (12.7)	61.6 (14.9)	0.394
Age > 70 years, *n* (%)	4 (14.3%)	10 (28.6%)	0.175
Male gender, *n* (%)	11 (39.3%)	21 (60%)	0.102
Underlying conditions			
Arterial hypertension, *n* (%)	8 (28.6%)	14 (40%)	0.344
Diabetes, *n* (%)	1 (3.6%)	6 (17.1%)	0.120
Chronic heart disease, *n* (%)	2 (7.1%)	1 (2.9%)	0.580
Chronic lung disease, *n* (%)	5 (17.9%)	6 (17.1%)	1
Chronic renal failure, *n* (%)	1 (3.6%)	8 (22.9%)	0.036
Recent bleeding (<30 days), *n* (%)	6 (21.4%)	2 (5.7%)	0.124
Risk factors for VTE			
Previous VTE, *n* (%)	6 (21.4%)	6 (17.1%)	0.667
Active cancer, *n* (%)	10 (35.7%)	6 (17.1%)	0.092
Recent surgery (<2 months), *n* (%)	13 (46.4%)	4 (11.4%)	0.002
High-risk PE, *n* (%)	16 (57.1%)	27 (77.1%)	0.090
Clinical presentation of PE, *n* (%)			0.013
Dyspnoea ± chest pain	19 (67.9%)	11 (31.4%)	
Syncope	6 (21.4%)	13 (37.1%)	
Cardiac arrest	3 (10.7%)	11 (31.4%)	

Abbreviations*:* PE, pulmonary embolism; VTE, venous thromboembolism.
